# Higher-Order Spike Triggered Analysis of Neural Oscillators

**DOI:** 10.1371/journal.pone.0050232

**Published:** 2012-11-30

**Authors:** Keisuke Ota, Toshiaki Omori, Hiroyoshi Miyakawa, Masato Okada, Toru Aonishi

**Affiliations:** 1 Brain Science Institute, RIKEN, Wako-shi, Saitama, Japan; 2 Department of Electrical and Electronic Engineering, Kobe University, Kobe-shi, Hyogo, Japan; 3 School of Life Sciences, Tokyo University of Pharmacy and Life Sciences, Hachioji, Tokyo, Japan; 4 Department of Complexity Science and Engineering, The University of Tokyo, Kashiwa-shi, Chiba, Japan; 5 Department of Computational Intelligence and Systems Science, Tokyo Institute of Technology, Yokohama-shi, Kanagawa, Japan; Georgia State University, United States of America

## Abstract

For the purpose of elucidating the neural coding process based on the neural excitability mechanism, researchers have recently investigated the relationship between neural dynamics and the spike triggered stimulus ensemble (STE). Ermentrout et al. analytically derived the relational equation between the phase response curve (PRC) and the spike triggered average (STA). The STA is the first cumulant of the STE. However, in order to understand the neural function as the encoder more explicitly, it is necessary to elucidate the relationship between the PRC and higher-order cumulants of the STE. In this paper, we give a general formulation to relate the PRC and the *n*th moment of the STE. By using this formulation, we derive a relational equation between the PRC and the spike triggered covariance (STC), which is the covariance of the STE. We show the effectiveness of the relational equation through numerical simulations and use the equation to identify the feature space of the rat hippocampal CA1 pyramidal neurons from their PRCs. Our result suggests that the hippocampal CA1 pyramidal neurons oscillating in the theta frequency range are commonly sensitive to inputs composed of theta and gamma frequency components.

## Introduction

A neural system can be considered to be an encoder which transforms specific external stimuli into neural spikes. One of the main goals of neuroscience is to identify the stimuli from the observed spikes. Spike triggered analysis is a powerful way to achieve this goal. In this analysis, we give stochastic stimuli to a neural system and identify the set of stimuli that induce the neurons to spike [1]. This set is called the spike triggered stimulus ensemble (STE) [2]–[4]. For example, the linear receptive field components in V1 simple cells can be discerned from the spike triggered average (STA), which is the average of STE [5], [6]. Additionally, the spike triggered covariance (STC) which is the covariance of the STE helps to clarify the receptive field structure of complex cells representing the nonlinear response [7], [8].

The traditional spike-triggered analysis with sensory stimuli can extract the receptive field properties of sensory neurons. Using direct current injection stimuli instead of sensory stimuli, the spike-triggered analysis can extract the set of presynaptic inputs encoded by individual neurons. The big advantage of the spike-triggered analysis with direct current injection stimuli is that it enables us to capture the coding properties of neurons in higher brain regions, such as the hippocampal regions, which are far away from the lower order sensory regions.

This spike triggered analysis can be formulated in terms of Bayes’ rule,
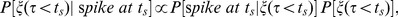
(1)where 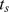 is spike time and 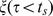 is a stimulus which has been applied to neurons before 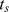. 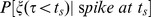 is the probability density function (PDF) of the STE; it is the conditional PDF of the stimulus 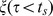 given the spike timing 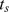. 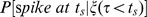 represents the spike generation process of the neural systems and is the PDF of the spike timing 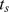 given the stimulus 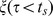, and 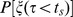 is the PDF of the stochastic stimulus applied to the neural systems. The PDF of the STE or statistics can be identified in two ways. One is the indirect way, in which 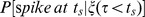 is determined and then 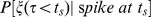 is obtained from Eq. (1). The other way is to obtain the distribution directly by measuring spike timings of neural systems in response to the stochastic stimulus 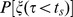. The direct way is almost always used for the spike triggered analysis in vivo experiments.

To clarify the relationship between neural dynamics and neural coding, several groups have recently tried to identify the STE statistics (i.e. STA and STC) in the indirect way [9]–[12]. Ermentrout et al. related neural dynamics to neural coding when regularly firing neurons are disturbed by sufficiently small white noise [13]. They analytically proved that the STA is proportional to the temporal differentiation of phase response curve (PRC), which represents an impulse response of an oscillatory system and captures the essence of the neural dynamics. Furthermore, they performed whole cell recording from olfactory bulb mitral cells and showed that their theory holds true for real cells. They made progress in relating the neural dynamics to the neural coding for real oscillating neurons. However, the STA is the first cumulant of the STE. To better understand the neural encoder function, it will be necessary to elucidate the relationship between the PRC and the higher-order moments of the STE.

In this paper, we focus on the neurons spiking almost periodically on the same assumption as Ermentrount et al. We propose a general formulation to relate the PRC and the *n*th moment of the STE (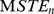) based on the Bayes’ rule in Eq. (1) [14]. In fact, we derive two relational equations. One relates the PRC to the STA, and the other relates the PRC to the STC. The relational equation between the STA and PRC coincides with the equation derived in [13]. This consistency shows that our formulation method includes Ermentrout et al.’s framework and is an extension of their theory. Additionally, the relational equation between the PRC and the STC allows us to determine the feature space, which is a low-dimensional subspace of the full stimulus space and characterizes the stimulus encoded by neurons [3], [10], [11]. We apply the relational equation to identifying the feature space of hippocampal CA1 pyramidal neurons oscillating in the theta frequency range (4–14 Hz) [15]–[17] from the estimated PRCs [18]. We show that the first principal component representing the feature space is the suppressive eigenfunction mainly composed of theta frequency components, whereas the second principal component is the excitatory eigenfunction mainly composed of gamma frequency components [19], [20].

## Methods

### Phase Description of Spike Generation Process

We will begin by describing the spike generation process of the oscillating neurons. Let us consider regularly firing neurons perturbed by sufficiently small white Gaussian noise stimulus. This neural behavior can be described by
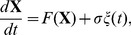
(2)where 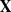 is the state of the neuron, 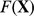 the vector field of neural dynamics, 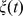 the white Gaussian noise stimulus, and 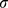 the stimulus intensity. 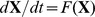 has a stable limit cycle solution 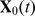 with period 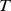. When the stimulus intensity is sufficiently small (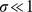), we can apply the phase reduction method [21] to Eq. (2) and obtain the following Langevin phase equation (LPE) [18], [22]–[25]:



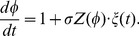
(3)Here, 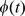 represents the phase defined along the limit cycle solution 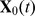 and 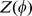 represents the PRC that corresponds to the impulse response function quantifying the phase response of the neural oscillator to the small perturbations. By introducing a slow phase variable 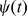 as 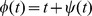, Eq. (3) can be transformed into
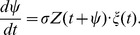
(4)Let 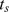 be the spike time after the first spike at 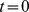. Since 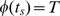, 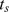 is given by
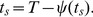
(5)By integrating both sides of Eq. (4) from 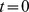 to 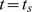, we can describe 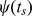 in terms of the PRC as follows:
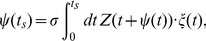
(6)where 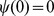. Since 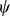 varies slowly when 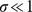, we can expand the right-hand side of Eq. (6) [23], [26]:



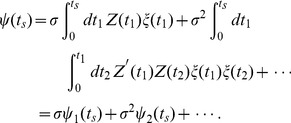
(7)This is now in the form of a Volterra series, which is widely used in analyzing oscillators driven by noise. Here, 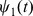 corresponds to a linear convolution term, which is derived under the assumption that 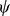 stops during a change of 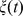. 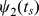 corresponds to the noise-induced drift in the Stratonovich definition [27]. It stems from the fact that 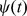 also changes when 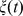 changes.

### General Formulation for the *n*th Moment of STE

Next, let us describe the *n*th moment of STE (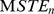). 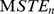 is defined as the *n*th time correlation of the current input 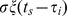, which represents the input preceding the spike time 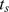 by 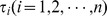. On the basis of Hong et al.’s formulation [14], 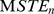 for the neural oscillators can be described with Bayes’ rule in Eq. (1):
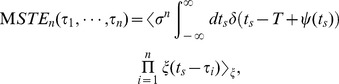
(8)where 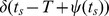 is the probability density of spike timings corresponding to 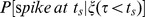 in Eq. (1) and 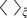 represents 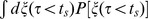. Because Eq. (8) represents integrals of the probability density of Eq. (1) over all stimulus inputs and all spike times 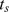, this equation gives 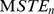 as the expected value of 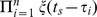 integrated over all spike times 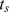.

Here, we shall denote the *m*-th order derivative of the delta function as 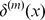, and write the Taylor expansion of the delta function as 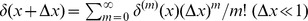, where 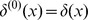 and 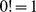. Since 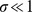, the right-hand side of Eq. (8) can be expanded as follows:
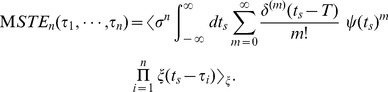
(9)This is the relational equation between the 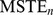 and the PRC, because 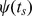 is represented by the PRC in Eq. (7).

### Relating STA and STC to the PRC

We can analytically derive the STA or the STC from Eq. (9) when 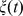 is zero-mean Gaussian white noise. In this case, higher correlation functions are given by 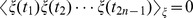 and 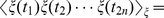 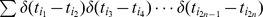. Here, the sum has to be performed over those 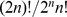 permutations in which 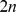 elements are separated into 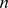 pairs [27]. The correlation of the raw stimulus input is
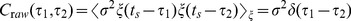
(10)We shall first derive an approximate equation relating the STA to the PRC. The STA is defined as the first moment of the STE: 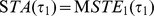. Substituting Eq. (7) into Eq. (9) and evaluating the lowest order, we obtain the approximated equation relating the STA to the PRC as



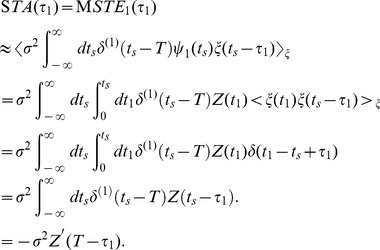
(11)

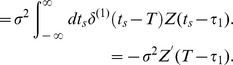


To obtain the last line, we used the delta function property, 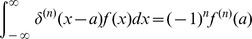. This result is consistent with the equation derived by Ermentrout et al. [13].

Next, we derive the approximated equation relating the STC to the PRC. The STC is defined as the second moment around the STA: 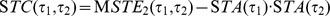. Similarly, substituting Eq. (7) into Eq. (9) and evaluating the 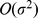 and 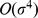, we obtain
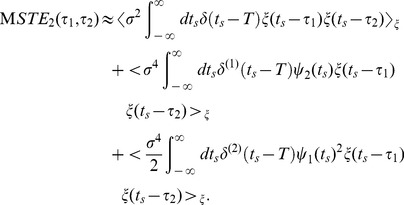
(12)


Note that the 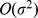 term of 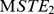 is equivalent to the variance of the raw stimulus ensemble 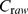 and the 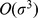 term disappears since 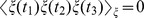; therefore, we should evaluate 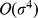 term including the fourth order correlation 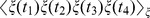. The second term in the right-hand side of Eq. (12) is
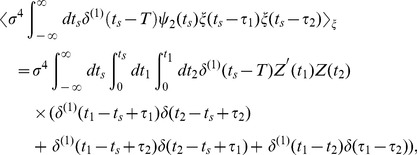
(13)and the third term is



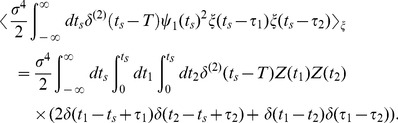
(14)By using the delta function property again, we finally obtain the approximate equation relating the STC to the PRC as follows:
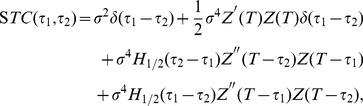
(15)where 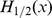 represents a Heaviside function which takes 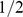 at 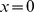.

### Feature Space Extraction

Although the input stimulus space is of high dimensionality, the feature space, which is spanned by the stimuli encoded by neurons, is a low-dimensional subspace of the full stimulus space. The feature space can be extracted by conducting an eigenvalue analysis of the difference between the STC and the correlation of the raw stimulus input:
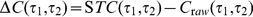
(16)The matrix of the STC represents the variance of a collection of samples in all possible directions within the space of spike-triggered stimuli, and 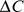 captures the relative change of the variance of the ensemble of input stimuli due to the rearrangement of stimuli in the time window preceding each spike. The eigenvalue of 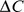, which is equal to the relative change of the variance in the direction of the corresponding eigenfuction, characterizes the sensitivity of neuron in response to the corresponding eigenfuction. The eigenfunctions with positive eigenvalues are referred to as the excitatory eigenfunctions, which enhance neural activity, whereas those with negative eigenvalues are called the suppressive eigenfunctions, which suppress neural activity. In the case of neural oscillators analyzed here, the stimuli in the subspace spanned by excitatory eigenfunctions cause shorter interspike intervals (ISIs) than the average period 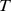, while the stimuli in the subspace spanned by suppressive eigenfunctions cause longer ISIs. Here, as in previous studies on spike triggered analyses for neural oscillators [28], we extract the feature space of neurons by computing the set of eigenfunctions with the large nonzero eigenvalues of 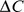 in absolute value.

### PRCs of Hippocampal CA1 Pyramidal Neurons

We used PRCs obtained from hippocampal CA1 pyramidal neurons by performing whole-cell patch-clamp recordings in vitro in our previous work [18]. In our protocol for measuring PRCs, we inject DC depolarizing currents into somata of CA1 pyramidal neurons to evoke periodic firing. Using the dynamic clamp, the mean ISI is adjusted to the target period of 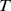 by tuning the DC depolarizing current. Next, a one-shot rectangle perturbation superimposed on the DC depolarizing current is evoked using various timings relative to the spike, and we measure how the perturbation current disturbs the timing of the succeeding spike. Spike times are randomly fluctuated by intrinsic noise in neurons. To extract PRCs from stochastic data of phase responses, we apply the maximum a posteriori (MAP) estimation algorithm that we proposed [29], [30] to the in vitro data. As described in the Numerical experiments section of the Methods, the notable feature of this algorithm is its use of a detailed PRC measurement model formulated as an LPE, which is the same as the one used in the current study. The effectiveness of the measurement model and the reliability of the estimated PRCs were verified by testing whether the LPEs with the estimated PRCs could predict the stochastic behaviors of the same neurons, whose PRCs had been measured, when they were perturbed by various periodic stimulus currents. Detailed explanations of experimental conditions and the MAP estimation algorithm can be found in [29]–[31].

### Numerical Experiments

#### Conductance-based model

To verify the algorithms by using numerical simulations, we use the Morris-Lecar (ML) model [32] in the form of
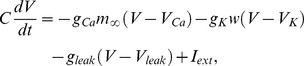
(17)where each ion channel has the following activation profile:



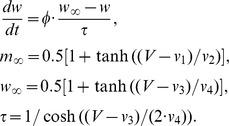
Depending on the parameters, the ML model has different bifurcation structures, classified as Type I and Type II [32]. In Type I model, oscillations emerge through a saddle-node bifurcation on an invariant circle. The parameters for the Type I model used in the simulations are 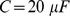, 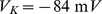, 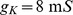, 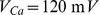, 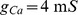, 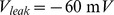, 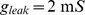, 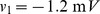, 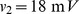, 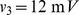, 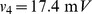 and 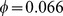. In the Type II model, oscillations emerge through a Hopf bifurcation. The parameters for the Type II model used in the simulations are 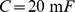, 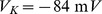, 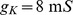, 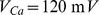, 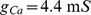, 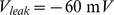, 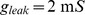, 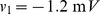, 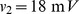, 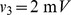, 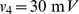 and 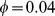.

The extra current 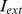 used in each numerical experiment is defined as follows.

#### Numerical calculation of STA and STC

By employing the Euler method, we numerically solved the ML model with the extra current 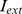 in the form of
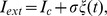
where 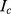 is the depolarizing constant current for inducing rhythmical firing with an average period 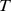. 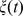 is the white Gaussian noise stimulus satisfying 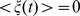 and 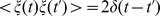. 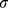 is the intensity of the stimulus used in the numerical simulations. In the Type I model, 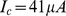 for 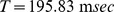 and 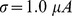. In the Type II model, 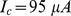 for 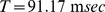 and 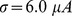.

In the numerical simulation, we measured the spike time, 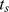 while we stored the noise stimulus 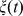. Here, we denote the time of the *i*th spike as 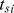, and the time sequence of the noise stimulus that were presented over an averaging interval T preceding the *i*th spike as 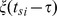, (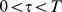).

We numerically calculated the first moment of STE, 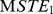 by taking the sample average of the stimuli [3], [8]:
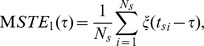
where 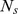 indicates the number of samples. As described above, the first moment of STE is called the STA. In a similar fashion, we numerically obtained the second moment of STE, 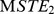 by calculating the covariance of the stimuli [3], [8]:



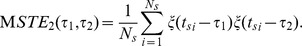
Then, according to the definition of the STC [3], [8], we can obtain the sample STC from the first and second moments:



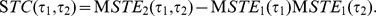



#### Adjoint method for calculating infinitesimal PRCs

We numerically calculated the infinitesimal PRCs of the ML model by using the adjoint method [21], [33]. In this numerical calculation, we used the extra current in the form of 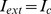 (i.e., without noise). In the same manner as above, 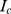 is the depolarizing constant current for inducing rhythmical firing with the period 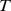. In the Type I model, 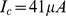 for 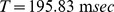, and in the Type II model, 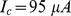 for 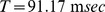.

Generally, the adjoint to the linearization of the unperturbed oscillator, 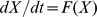 in Eq. (2) on a limit cycle orbit 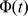 satisfies:
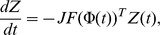
where 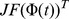 is the transpose of the Jacobi matrix of 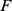 on the orbit. Due to the stability of the system in the orbit, the Jacobi matrix 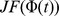 only has nonpositive eigenvalues. Hence, the above adjoint system is unstable, and it has an unstable limit cycle orbit. This unstable limit cycle orbit corresponds to the infinitesimal PRCs. By reversing the time in the numerical calculation, we can stably obtain the unstable limit cycle orbit.

#### Estimation of PRCs from artificial data

To check the applicability of the theory to real neurons, we used a PRC estimated from a finite sample of artificial phase responses generated with the Type I ML model. In the numerical simulation for generating artificial phase response data, we used an extra current 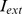 in the form of
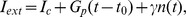
where 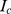 is the depolarizing constant current for inducing rhythmical firing with an average period 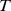, and 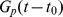 is the rectangle pulse current whose 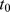 represents the timing of its appearance. 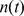 is white Gaussian noise satisfying 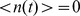 and 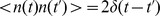, and 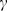 is the intensity of the noise. The parameters we used are 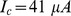 for 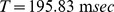, 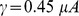, amplitude of 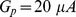, and duration of 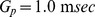.

By employing the Euler method, the phase response were sampled as a sequence of equidistant points, 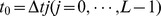, whose sampling period was 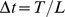, and 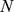 samples of phase responses were measured at each sampling point 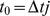. Thus, the total number of data was 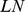. In the numerical experiment of this paper, 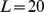 and 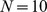.

We used the maximum a posteriori (MAP) estimation algorithm that we proposed in our previous papers to estimate the PRCs from the artificial data. The notable feature of this algorithm is its use of a detailed PRC measurement model formulated as the LPE, which is the same as the one used in this work. Moreover, our algorithm enables one to estimate hyperparameters including the smoothness of the PRCs, whereas in previous studies, the smoothness was selected in an ad-hoc way. A detailed explanation of the MAP estimation algorithm can be found in [29], [30].

## Results

### Simulation

**Figure 1:**
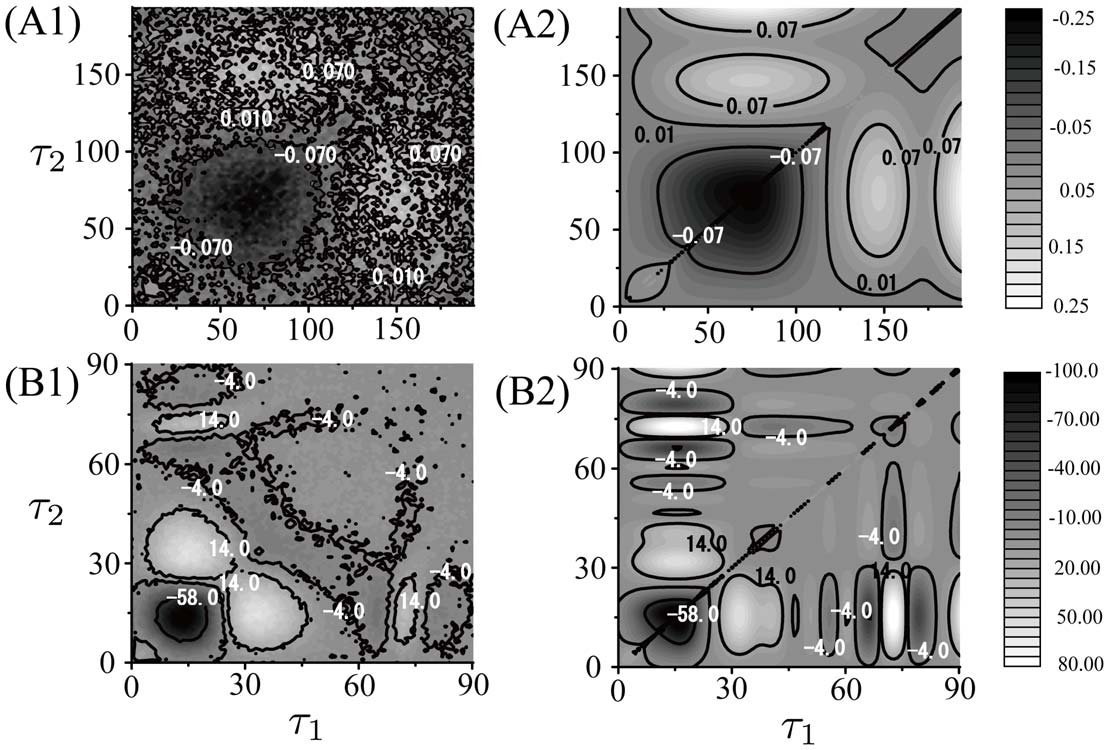
Numerically simulated STC (A1, B1) and theoretically derived STC (A2, B2). Here the delta-peak at the point 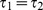 is replaced with the average of the nearest matrix elements values. (A) Type I ML model, 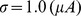. (B) Type II ML model, 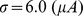. The DC input current used in (A) and (B) is 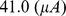 and 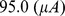 respectively, which induces regular spikes with intervals of about 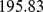 msec and 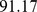 msec. In all the experiments, the numerically simulated STC were computed using a sample of 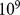 spikes and the matrix size of the STC was 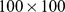.

In order to confirm our theory, we compared the STC calculated from Eq. (15) and the STC obtained in a numerical experiment. Figure 1 (A1) and (B1) show the numerically simulated STCs for Type I and Type II Morris-Lecar (ML) model [32], respectively. Figure 1 (A2) and (B2) illustrate the analytically derived STCs, where the PRCs for each ML model are derived with the adjoint method [21], [33]. These analytically derived STCs are in good agreement with the numerically simulated ones. This result indicates that the STC can be computed accurately with our theory.

**Figure 2:**
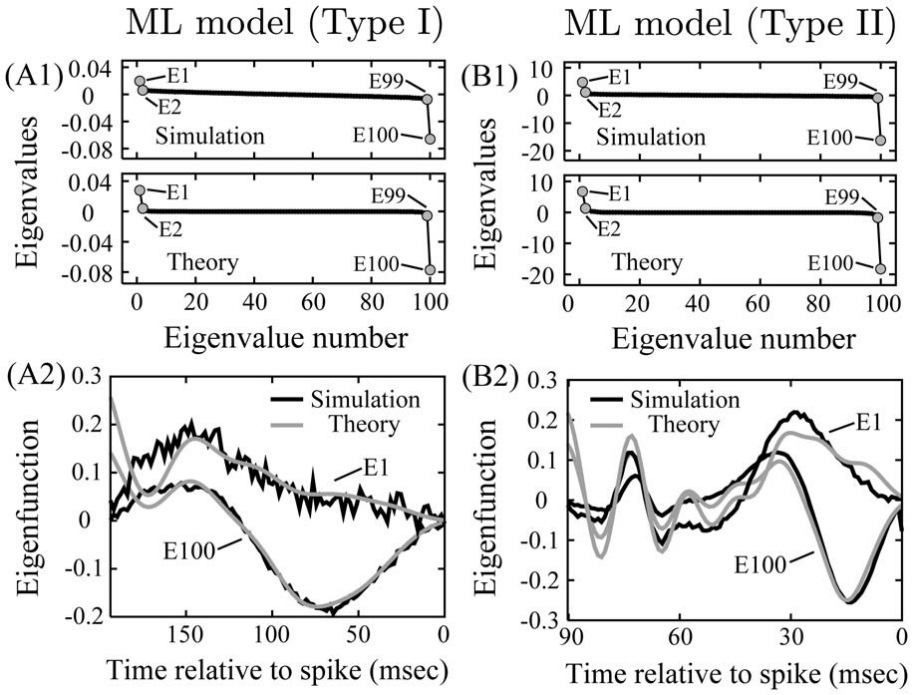
Eigenvalue analyses of the theoretically derived 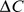 and numerical simulation. (A1, B1) The eigenvalue spectrum of the numerically simulated 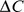 (upper) and the eigenvalue spectrum of the theoretically obtained 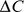 (bottom). (A2, B2) The eigenfunctions associated with the maximal eigenvalue (E1) and minimal eigenvalues (E100). The eigenfunctions derived from the theoretically obtained 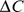 (gray line) mostly match those derived from the numerically simulated 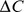 (black line). This eigenvalue analysis is independent of the matrix size of 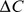.

We conducted an eigenvalue analysis of the neural oscillators. Figure 2 compares the eigenvalue analyses of the theoretically derived 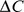 and numerical simulation. The theoretically derived eigenfunctions with the maximal and minimal eigenvalues (E1 and E100) are consistent with the numerically simulated eigenfunctions (Fig. 2 (A2) and (B2)). The theoretical result also matches the numerical results for the eigenfunctions with second maximal and minimal eigenvalues (E2 and E99) (data not shown). These results suggest that our theory can identify the neural feature space of neurons firing periodically if we know only the PRC.

**Figure 3:**
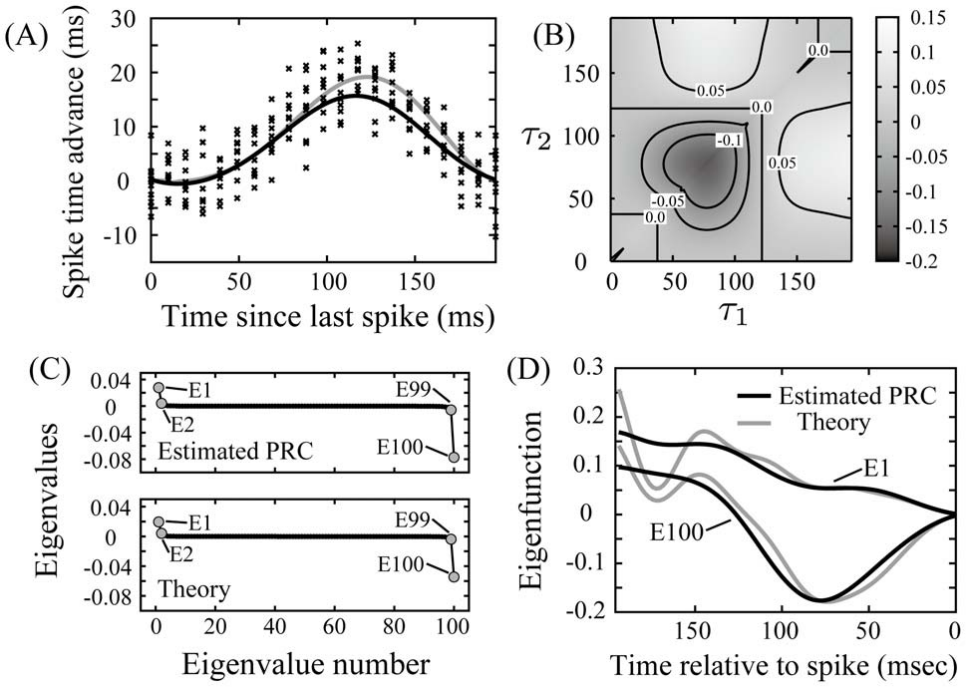
The STC, the eigenvalue spectrum and the eigenfunctions calculated with the PRC estimated from a finite number of artificial noisy data. (A) Crosses show artificial phase response data generated with the Type I ML model. 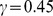, 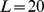 and 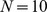. The gray solid line is the PRC derived with the adjoint method, and the black solid line indicates an estimated PRC as a result of applying the MAP estimation algorithm to the artificial data. (B) STC calculated from the estimated PRC with the artificial data. 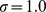. (C) Upper: the eigenvalue spectrum of 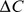 calculated from the estimated PRC with finite noisy samples. Lower: one calculated from the PRC derived with the adjoint method, which is the same as the lower part of Fig. 2(A1). (D) The eigenfunctions associated with the maximum and the minimal eigenvalues, E1 and E100. Black solid lines are calculated with the estimated PRC. Gray solid lines are calculated with the PRC with the adjoint method, which are the same as the gray solid lines of Fig. 2(A2).

When applying the theory to extracting the feature space of real neurons, we have to use the PRCs estimated from a finite sample of noisy phase responses measured in vitro. Here, we verified how estimation errors of the PRCs affect the reliability of the STCs. We numerically generated artificial noisy phase response data using the Type I ML model, and we estimated the PRC from the artificial data with the maximum a posteriori (MAP) estimation algorithm that we previously proposed [29]. After that, we compared the STC, the eigenvalue spectra and the eigenfunctions calculated from the estimated PRC with those from the PRC derived with the adjoint method (Fig. 1(A2) and Figs. 3(B)-(D)). As shown in Fig. 3(A), the estimated PRC with finite noisy samples conforms to the PRC derived with the adjoint method in the first half of the period, but is different in the second half of the period. As shown in Figs. 1(A2), 3(B) and 3(D), the STC and the eigenfunctions calculated with the estimated PRC are in good agreement with ones derived by the adjoint method at earlier times relative to the spike, but they have few differences at later times relative to the spike.

### Feature Space of the Hippocampal CA1 Pyramidal Neurons

**Figure 4:**
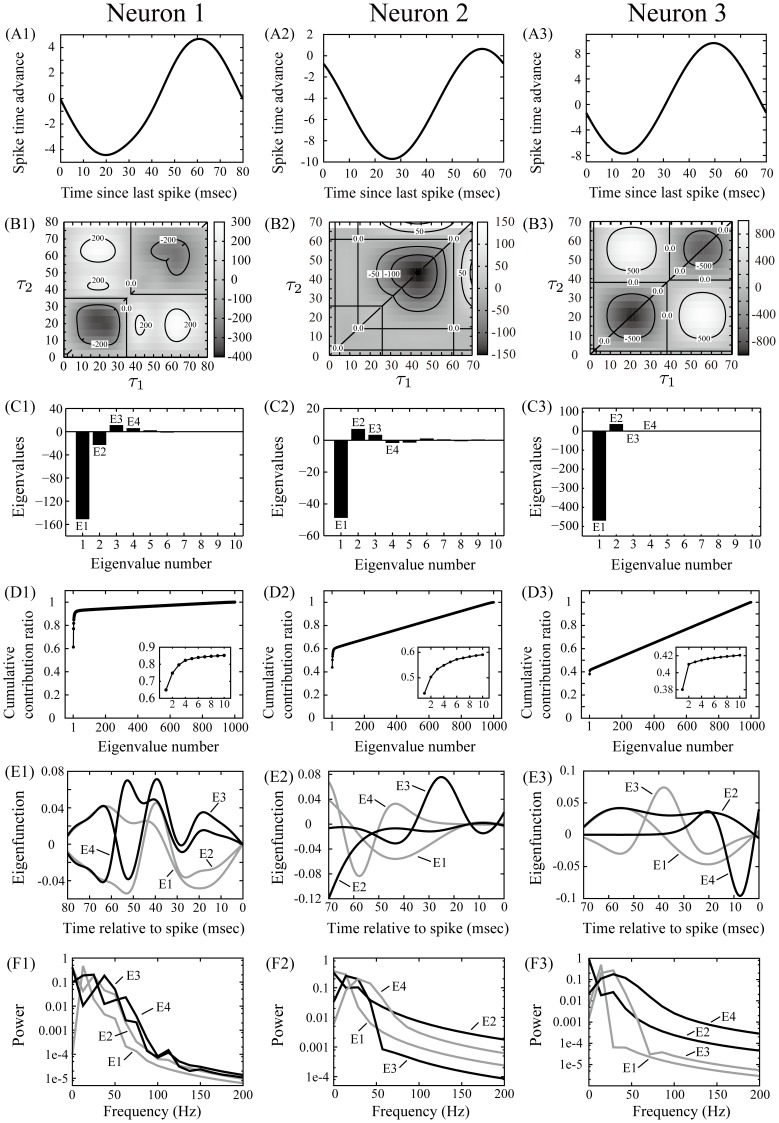
PRCs, STCs and eigenvalue analysis results for 3 rat hippocampal CA1 pyramidal neurons. (A) PRCs of 3 rat hippocampal CA1 pyramidal neurons [18]. The firing period is (A1) 80 msec and (A2, A3) 70 msec. (B) The STCs for PRCs shown in (A). It is calculated using Eq. (15) into which we substitute the estimated PRC and noise intensity. The matrix size is 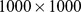. (C) The eigenvalue spectra of 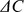 arranged in order of absolute magnitude. Unlike Fig. 2, the number on the eigenvalues in Fig. 3 is assigned in order of absolute magnitude. (D) The cumulative contribution ratio of the magnitude of the eigenvalues. (E) The eigenfunctions for eigenvalues E1, E2, E3 and E4. (F) The power spectra of the top 4 eigenfunctions shown in (E).

We identified the feature space of rat hippocampal CA1 pyramidal neurons oscillating in the theta frequency range (4–14 Hz) [15]–[17]. Figure 4 (A) shows the PRCs of pyramidal neurons, which have been measured in vitro in previous works [18], [30], [31].

Figure 4 (B) shows the STCs calculated from the PRCs and Fig. 4 (C) the eigenvalue spectra for the STCs arranged in order of absolute magnitude. Note that it was impossible to accurately distinguish between a zero and nonzero eigenvalue because of the numerical precision limitation. Instead, we evaluated the cumulative contribution ratio of the magnitude of the eigenvalues (Fig. 4 (D)). Figure 4 (E) shows the top 4 eigenfunctions highly contributing to the representation of the feature spaces for each neuron. Those phasic relationships to the neuron firing (i.e., positive and negative parts of the eigenfunctions) are different from each other. Here, to elucidate the common characteristics of those eigenfunctions representing highly sensitive inputs of those neurons, we calculated power spectra of the eigenfunctions (Fig. 4(F)).

Here we checked a total of five samples including two other samples of pyramidal neurons not shown in Fig. 4. E1 in all five samples, including the three samples in Fig. 4, has negative eigenvalues, and the power spectra of E1 in all samples except for neuron 2 have peaks in the theta frequency range. For neuron 2, the power spectrum of E1 has a peak at zero frequency and is relatively large in the theta frequency range. On the other hand, E2 in all samples except for neuron 1 has positive eigenvalues, and the power spectra of E2 in all of five samples have the first peaks at zero frequency and second peaks in the gamma frequency range (20–80 Hz). Therefore, we can conclude that the first principal component of the feature space is the suppressive eigenfunction mainly composed of theta frequency components, whereas the second principal component is the excitatory eigenfunction mainly composed of DC and gamma frequency components. This result suggests that theta waves suppress neural activity, leading to longer ISIs than the average period 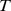, whereas DC and gamma waves enhance neural activity, leading to shorter ISIs.

## Discussion

In this study, we proposed the general formulation to relate the PRC and the 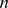
th moment of the STE. At first, using the new formulation, we analytically derived approximate equations that relate the PRC to the STA. The relational equation between the STA and PRC was equal to the equation derived by Ermentrout et al. [13]. Thus, our formulation method includes their theory. Next, using the formulation, we were able to successfully derive the relational equation between the PRC and the STC. This relational equation allows us to determine the feature space only from the PRC.

We used the relational equation to identify the feature space of the rat hippocampal CA1 pyramidal neurons oscillating in the theta frequency range. We showed that the first principal component representing the feature space is the suppressive eigenfunction mainly composed of theta frequency components, whereas the second principal component is the excitatory eigenfunction mainly composed of DC and gamma frequency components. This result suggests that the hippocampal CA1 pyramidal neurons oscillating in the theta frequency range are commonly sensitive to inputs composed of DC, theta, and gamma frequency components. Theta waves prolong the ISIs of theta oscillating neurons whereas DC and gamma waves shorten them. Note that the DC sensitivity is trivial because an increase in the depolarization current shortens the ISIs. Therefore, our results imply that during theta oscillation the ISIs of the CA1 pyramidal neurons can be modulated by inputs oscillating at theta and gamma frequencies. Several studies have observed the theta oscillation in the CA1 area of the hippocampus in freely behaving rats and have found that the gamma waves are superimposed on the theta oscillation [34], and many researchers have focused on interactions between theta and gamma oscillations in an attempt to account for the temporal coding of the hippocampus [35]. Our finding suggests that interactions between theta and gamma oscillations can be realized at the single neuron level.

The shape of PRCs shown in Fig. 4 is different from those measured by Netoff et al [36]. This is because the periods of oscillatory activates we tuned in measuring PRCs are slightly shorter than 100 ms intervals they used. It is well known that the shape of PRCs strongly depends on the period of the oscillation [37].

As expressed in Eqs. (7) and (8), we ignored the refractoriness in the formulation for the moment of STE. Using Omori et al.’s formulation [12], we can deal with the refractory effect, because this formulation enables us to prevent miscounting events in which the spike is reset after a spike to just prior to a spike.

Small excitatory inputs applied at the end of the phase cannot advance the action potential to a time prior to the application of the stimulus. This causality limit changes the noise characteristics of a measured PRC at the end of the phase even when no dynamical change may occur. However, the LPE we used to describe stochastic fluctuations of phase responses in the MAP estimation algorithm does not have a resetting mechanism immediately after spike generation as in the integrate-and-fire neuron model, so this algorithm cannot deal with the causality limit problem [18], . One approach to account for this effect to estimate the phase advance where the mean is estimated using a truncated Gaussian distribution to describe stochastic fluctuations of phase responses [38].

We obtained the STC shown in Figs. 1 (A1) and (B1) from lots of spikes. The number of neural spikes required for a stable calculation of the STC is nearly the square of the number required for the STA. Ermentrout et al. calculated the STA of the olfactory bulb mitral cell from several thousand spikes [13]; therefore, the STC requires more than 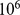 spikes, which is much more than what would be available in a physiological experiment. On the other hand, the PRCs have been measured from just several hundred spikes in several real neurons [18], [39]–[43]. Equation (15) enables us to obtain the STC of the real neuron through the PRC.

The PRCs of the hippocampal CA1 pyramidal neurons recorded in vitro used here are defined as changes in ISI between two successive single spikes in response to small perturbations for regularly firing neurons. However, temporal spike patterns of the hippocampal CA1 pyramidal neurons in vivo show more bursty behaviors than those we assume here. Hence, our assumption does not correspond to actual behaviors very well. Despite this, our theory can be straightforwardly applied to bursty situations. For bursty neurons, the burst phase response curves (BPRCs), which are defined as changes in the interval between two successive bursts in response to small perturbations, can be obtained by computing with an infinitesimal perturbation approximation and by directly stimulating the neurons, as we did [44]–[46]. By using BPRCs instead of PRCs, one can expect to capture coding properties closer to those of in vivo situations.

To make in vitro experiments for measuring the PRC look like in vivo as much as possible, Lengyel et al. injected sinusoidal inhibitory conductance with the dynamic clamp, which mimics hippocampal theta oscillation, into somata of CA3 pyramidal neurons [40]. In contrast, we injected DC depolarizing currents into somata of CA1 pyramidal neurons to evoke periodic firing. By using the PRCs under external periodic perturbation, one can expect to capture coding properties closer to those of in vivo situations.

As described in Methods section, depending on bifurcation structures, neural oscillators can be classified as Type I and Type II [47]. It has been proved that near a bifurcation point, the infinitesimal PRC of Type I neuron only has positive values, whereas the infinitesimal PRC of Type II neuron has positive and negative (biphasic) values [33]. Thus, purely positive PRCs are habitually called Type I PRCs, whereas biphasic PRCs are called Type II PRCs. In line with this argument, Steifiel et al. showed physiologically and numerically that cholinergic action, which causes the down-regulation of slow voltage-dependent potassium currents such as the M-current, could switch the PRC from Type II to Type I [48], [49]. This result suggests that cholinergic modulation may change the bifurcation structure of neural dynamics, resulting in a qualitative switch of the PRCs type. If this suggestion is true, cholinergic modulation may cause a change in the feature space of neurons reflected in a qualitative switch of the PRCs type as shown in Figs. 1 and 2. From the results of Stiefel et al and our theory, there arises a possibility that we can do a longitudinal study of a neural system from the molecular level to the computational level.
